# Negotiating Discourses of Shame, Secrecy, and Silence: Migrant and Refugee Women’s Experiences of Sexual Embodiment

**DOI:** 10.1007/s10508-016-0898-9

**Published:** 2017-01-12

**Authors:** Jane M. Ussher, Janette Perz, Christine Metusela, Alexandra J. Hawkey, Marina Morrow, Renu Narchal, Jane Estoesta

**Affiliations:** 1 Centre for Health Research, School of Medicine, Western Sydney University, Sydney, NSW 2751 Australia; 2 Centre for the Study of Gender, Social Inequities and Mental Health, Simon Fraser University, Vancouver, BC Canada; 3 Family Planning New South Wales, Sydney, NSW Australia

**Keywords:** Women’s sexual embodiment, Migrant and refugee sexual health, Intersectionality, Sexual shame, Sexual health

## Abstract

In Australia and Canada, the sexual health needs of migrant and refugee women have been of increasing concern, because of their underutilization of sexual health services and higher rate of sexual health problems. Previous research on migrant women’s sexual health has focused on their higher risk of difficulties, or barriers to service use, rather than their construction or understanding of sexuality and sexual health, which may influence service use and outcomes. Further, few studies of migrant and refugee women pay attention to the overlapping role of culture, gender, class, and ethnicity in women’s understanding of sexual health. This qualitative study used an intersectional framework to explore experiences and constructions of sexual embodiment among 169 migrant and refugee women recently resettled in Sydney, Australia and Vancouver, Canada, from Afghanistan, Iraq, Somalia, South Sudan, Sudan, Sri Lanka, India, and South America, utilizing a combination of individual interviews and focus groups. Across all of the cultural groups, participants described a discourse of shame, associated with silence and secrecy, as the dominant cultural and religious construction of women’s sexual embodiment. This was evident in constructions of menarche and menstruation, the embodied experience that signifies the transformation of a girl into a sexual woman; constructions of sexuality, including sexual knowledge and communication, premarital virginity, sexual pain, desire, and consent; and absence of agency in fertility control and sexual health. Women were not passive in relation to a discourse of sexual shame; a number demonstrated active resistance and negotiation in order to achieve a degree of sexual agency, yet also maintain cultural and religious identity. Identifying migrant and refugee women’s experiences and constructions of sexual embodiment are essential for understanding sexual subjectivity, and provision of culturally safe sexual health information in order to improve well-being and facilitate sexual agency.

## Introduction

Sexual embodiment refers to the experience of living in, perceiving, and experiencing the world from the location of our sexual bodies (Tolman, Bowman, & Fahs, 2014). Women’s experience of sexual embodiment is located in the historical and cultural context in which they live (Jackson & Scott, 2007). This does not deny the material reality of the sexual body—but conceptualizes embodiment in a social context, reflexively constructed and reconstructed through sexual and social interactions (Jackson & Scott, 2002), which influences both how we see and experience our own and others’ sexual bodies (Ussher, 1997b). Discursive constructions of normal sexual experience and expression, mediated by factors such as race, social class, and cultural background, determine the sexual scripts which women are allowed to adopt, as well as the possibilities for resistance (Tiefer, 2004). Discursive constructions of sex and the meaning of the sexual body also have implications for sexual health and sexual subjectivity—a woman’s experience of herself as a sexual being, her feeling of entitlement to sexual pleasure and sexual safety, her ability to make active sexual choices, and her identity as a sexual being (Tolman, 2002). Within this “material-discursive” theoretical standpoint (Ussher, 1997a, 2008), discourses constitute systems of thoughts composed of ideas, attitudes, courses of action, beliefs and practices, that “systematically construct the subjects and the worlds of which they speak” (Foucault, 1972, p. 49).

In the English-speaking Western world, including North America, the UK, and Australasia, dominant discourses surrounding women’s sexuality have traditionally been tied to white, middle- and upper-class norms which dictate that “good” women engage in self-policing to contain and control their sexual desires (Fine & McClelland, 2006; Jackson & Lyons, 2013; Tolman, 2002). Acting as passive sexual gatekeepers within heterosexual relationships (Gagnon, 1990), women were not expected to know about sex, as they “simply need to say *no*” (Curtin, Ward, Merriwether, & Caruthers, 2011, p. 50). However, social constructionist and feminist theorists have argued that women have the potential to ‘rewrite’ or resist traditional constructions of sexuality through the mobilization of counter-stories that position their sexuality in more agentic ways (Day, Johnson, Milnes, & Rickett, 2010; McKenzie-Mohr & Lafrance, 2014). This is evident in young women’s adoption of “raunch culture,” discursive representations of a “sexually liberated, savvy and active woman who is “up for it” in terms of sex (Evans, Riley, & Shankar, 2010, p. 115). It is also evident in the widespread acceptance of a human rights-based sexual health discourse promoted by international governing bodies (United Nations, 2014), wherein “the sexual rights of all persons… are respected, protected and fulfilled” (World Health Organization, 2016). This rights-based sexual health discourse serves to legitimate women’s sexual agency—their ability to make free choices about their sexual expression (Corrêa, Petchesky, & Parker, 2008).

However, processes of patriarchal and heterosexist cultural, religious and familial power serve to create limitations to ways in which women can construct themselves and their options for resistance (Frosh, Phoenix, & Pattman, 2003). Women who embrace “raunch culture” and sexual agency may be vulnerable to social condemnation (Bishop, 2012), with agentic female sexuality positioned as sexual “promiscuity” and the woman herself at risk of being described as a “slag” (Bale, 2011; Jackson & Lyons, 2013). Despite this, there is evidence that many Western women negotiate competing constructions of feminine sexuality in order to attain positive and agentic sexual subjectivity (Bishop, 2012; Gavey & McPhillips, 1999) and sexual health (Bale, 2011; Stewart, 1999). This negotiation is analogous to what McKenzie-Mohr and Lafrance (2011) call “tight-rope talk,” wherein women construct themselves as “both active and acted upon” (p. 64), taking credit for sexual agency at the same time as maintaining the cultural capital that accrues from adhering to dominant discourses of feminine (hetero)sexual embodiment. For women who migrate to the West from cultures where a discourse of women’s sexual agency is less common (Rogers & Earnest, 2015), and where deeply entrenched social and cultural discrimination may create barriers to women’s sexual health (United Nations, 2014) this negotiation may be a more difficult and complex process. In this vein, previous researchers have argued that there is a need for research on how culturally and linguistically diverse (CALD)^1^ migrant and refugee women negotiate diverse discourses and cultural constraints associated with sexual embodiment (Rogers & Earnest, 2015; Sargent, 2006), in order to understand their sexual subjectivity and facilitate their sexual health. This is the aim of the present study.

Examining how migrant and refugee women negotiate discursive constructions of sexuality and sexual embodiment is important for a number of reasons. One of the outcomes of the adoption of a rights-based sexual health discourse in the West has been the widespread availability of sexual and reproductive health services for women, and sexuality education for young people, with positive implications for quality of life, mental health, and sexual well-being (Aggleton & Campbell, 2000; Chen, Subramanian, Acevedo-Garcia, & Kawachi, 2005; Stephenson et al., 2008). However, in Australia and Canada, the sexual health needs of migrant and refugee women has been of increasing concern, because of their underutilization of sexual health services (Botfield, Newman, & Zwi, 2016; Manderson & Allotey, 2003; McMullin, De Alba, Chávez, & Hubbell, 2005; Robinson et al., 2011). This has a number of consequences. Young migrant and refugee women may be ill equipped to articulate their sexual rights (Martinez & Phillips, 2008), having little knowledge of, or access to, preventative sexual health measures (Salad, Verdonk, de Boer, & Abma, 2015) or contraception (Ngum Chi Watts, Liamputtong, & Carolan, 2014). Absence of education about sexually transmitted infections (STIs) and fertility control can lead to information being sought from unreliable sources, such as peer groups or the media (McMichael & Gifford, 2009; Rawson & Liamputtong, 2009), as well as engagement in risky behaviors (Rawson & Liamputtong, 2010). Delayed testing or screening may result in late diagnosis and treatment of cervical cancer (Manderson & Allotey, 2003; McMullin et al., 2005), HIV, or STIs (Fenton, 2001). Inadequate contraception can lead to unplanned pregnancy, or family size that is not desired (Rademakers, Mouthaan, & Neef, 2005), with negative psychosocial implications for women (Bunevicius et al., 2009; Tsui, McDonald-Mosley, & Burke, 2010).

Specific cultural factors, combined with patriarchal family structures, may present barriers to accessing positive sexual embodiment and access to sexual health services following migration. Some cultural groups avoid all discussion of reproductive and sexual health, as it is viewed as too sensitive or disrespectful (Beck, Majumdar, Estcourt, & Petrak, 2005; Rawson & Liamputtong, 2010), resulting in health concerns not being addressed with partners, family members, or health providers. At the same time, sexual health services may be seen as culturally inappropriate (Allotey, Manderson, Baho, & Demian, 2004; Guerin, Allotey, Elmi, & Baho, 2006; Richters & Khoei, 2008), or may not match cultural constructions of good treatment (Manderson & Allotey, 2003), and thus be avoided. Cultural constructions about the etiology of illness, or the impact of interventions, may also inhibit access to sexual health services (Gagnon, Merry, & Robinson, 2002; Manderson & Allotey, 2003). For example, young migrant women have been reported to avoid emergency contraception because of the misperception that it is an abortifacient, which affects long-term health and fertility (Shoveller, Chabot, Soon, & Levine, 2007).

Culturally prescribed gender roles combined with patriarchal values can also influence a woman’s ability to negotiate safer sex practices within relationships (Gifford, Bakopanos, Dawson, & Yesilyurt, 1998; Ussher et al., 2012), making women more vulnerable to STIs and unplanned pregnancy (Gifford et al., 1998). A discourse of marital sexual duty can lead to women focusing on the sexual needs of their husband (Khoei, Whelan, & Cohen, 2008), or enduring marital rape (Boonzaier & de La Rey, 2003), resulting in the absence of sexual agency and significant levels of distress (Martin, Taft, & Resick, 2007). It has been reported that many women from immigrant and refugee communities think it is inappropriate and disrespectful to raise issues of sexual health within a relationship, and some married women feared their husbands would divorce them if they insisted on safer sex (Gifford et al., 1998), or if they contracted a sexually transmitted disease (Go et al., 2002). Cultural shame and stigma can act as barriers to positive sexual embodiment and access to sexual health services (de Anstiss & Ziaian, 2010; McMichael & Gifford, 2009), sometimes reinforced by stigmatizing messages from health providers (Shoveller et al., 2007), resulting in avoidance of health care and information being sought from informal sources instead (de Anstiss & Ziaian, 2010; Manderson, Kelaher, Woelz-Stirling, Kaplan, & Greene, 2002; Rawson & Liamputtong, 2010). Within cultures that emphasize the importance of virginity, sexual health services may be seen as inappropriate for young and unmarried women (Beck et al., 2005; McMullin et al., 2005), with personal reputation or family honor jeopardized if it is known women are engaging in premarital sex (Manderson et al., 2002; Rawson & Liamputtong, 2010). This can result in young women being ill equipped to articulate their sexual rights (Martinez & Phillips, 2008), having little knowledge of, or access to, contraception, and social exclusion if pregnancy occurs outside marriage (Ussher et al., 2012).

Cultural shame associated with menarche and menstruation (Cooper & Koch, 2007; Sommer, Ackatia-Armah, Connolly, & Smiles, 2015), premarital sex (Kebede, Hilden, & Middelthon, 2014; Meldrum, Liamputtong, & Wollersheim, 2014), discussion of sexuality (Dean, Mitchell, Stewart, & Debattista, 2017; Quelopana & Alcalde, 2014), STIs (McMichael & Gifford, 2009), HPV inoculation and cervical cancer screening (Salad et al., 2015), and contraception use (Ngum Chi Watts et al., 2014; Sargent, 2006), may also influence women’s sexual embodiment. This shame needs to be conceptualized in the context of broader cultural and historical constructions of women’s sexual and reproductive bodies as dirty or polluted (Douglas, 1966), as a sign of abjection (Kristeva, 1982), described as the “monstrous feminine” (Ussher, 2006). This suggests that while there may be conflict between sexual discourses and norms in migrant and refugee women’s country of origin and country of settlement (Meldrum et al., 2014; Wray, Ussher, & Perz, 2014), there are also commonalities across cultures.

One of the limitations of previous research in this field is that it has not examined commonalities or differences in constructions or experiences of the different aspects of sexuality and sexual health outlined above, leading to a potential fragmentation of research findings into specific topic areas, such as menstruation, contraception, or sexual health screening. There has also been a focus on experiences of young unmarried women (e.g., McMichael & Gifford, 2009; Rawson & Liamputtong, 2010; Wray et al., 2014), with only a minority of studies examining adult women’s sexual health (e.g., Richters & Khoei, 2008; Ussher et al., 2012), leading to a call for research on adult women and those who are parents (Keygnaert, Vettenburg, Roelens, & Temmerman, 2014; McMichael & Gifford, 2009). Equally, while there has been documentation of conflict between sexual discourses and norms in women’s country of origin and country of settlement (Meldrum et al., 2014; Wray et al., 2014), little attention has been paid to women’s negotiation of this conflict. And while researchers have begun to examine sexual embodiment in other areas of sexuality studies (e.g., Duncan, 2010; Jackson & Scott, 2001, 2002; Tolman et al., 2014), a focus on “the experience of living in, perceiving, and experiencing the world from the very specific location of our bodies” (Tolman et al., 2014, p. 760) has been absent from research on migrant women’s sexual health.

The focus of sexual and reproductive health research has also been on single cultural groups, primarily Vietnamese or South-East Asian populations (Gagnon et al., 2002; Garrett, Dickson, Whelan, & Whyte, 2010), which results in the marginalization or invisibility of sexual health experiences and needs across a range of migrant groups (Garrett et al., 2010). If more than one cultural group is included, migrants are invariably treated as a homogenous population, which negates variations which occur within and between cultures (Beiser, 2005; Rawson & Liamputtong, 2010), the complexity of culture as a concept (Morrow, Smith, Lai, & Jaswal, 2008), as well as the intersections of cultural background with other social structuring factors, such as age, religion, or geographical location (Botfield et al., 2016; Manderson & Allotey, 2003; Martinez & Phillips, 2008). There is a need for further research to understand the intersecting influences of culture, age, religion, geography, migratory experience, and relationship status on women’s sexual embodiment.

The aim of this study was to address these gaps in the research literature, through examining the negotiation of discursive construction of sexual embodiment, across a range of experiences, in adult migrant and refugee women from a number of cultural groups, living in Sydney, Australia, and Vancouver, Canada. The research questions were: 1. How do migrant and refugee women discursively construct their sexual embodiment—focusing on the processes by which cultural meanings associated with sexual embodiment are produced and understood by women? 2. How do women negotiate competing discourses associated with sexuality and sexual embodiment? 3. What are the implications of these constructions and negotiations for sexual health knowledge and behavior? Our questions were meant to explore women’s experiences of sexual embodiment and also the ways in which intersecting forms of oppression (especially sexism and heterosexism) might form structural barriers related to accessing sexual health information.

## Method

### Participants

Eighty-four one-to-one interviews and 16 focus groups comprised of 85 participants were conducted (total *n* = 169) from July 2014 to March 2016. The majority of participants (73%, *n* = 124) were interviewed in their first language by community interviewers, women from the same cultural background as the interviewees. Women who preferred to speak English, or who could speak English and preferred to be interviewed by a non-community member, were interviewed by a member of the research team, in five focus groups (*n* = 16) and 29 one-to-one interviews.

Participants were migrant and refugee women 18 years and over who had settled in Australia or Canada in the last 10 years. Participants originated from various countries, including Afghanistan, Iraq, Somalia and Sudan. Sri Lankan (Tamil), Indian (Punjab) and South Sudanese women were also included in the Australian sample and women from various South American (Latina) backgrounds in the Canadian sample, to allow for analysis of sexual embodiment within and across cultural groups. Women identified as practicing a range of religions, including Islam, Christianity, Sikhism and Hinduism, and encompassed a range of social class backgrounds within their country of origin. All participants, except for one Latina woman, identified as being heterosexual and ranged from 18 to 70 years old, with a mean age of 35. The average length of time since migration was 6.3 years, with the majority of women migrating as humanitarian refugees. The exceptions were Punjabi participants in Australia who migrated as skilled migrants or for family reunion, and the Latina women who migrated as farm workers. Recruitment within cultural groups continued until saturation of data was achieved—no new information in three consecutive interviews. Table 1 provides the demographic information for the sample.

**Table 1:** Demographics for participant interviews and focus groups by ethnicity

	Afghani	Iraqi	Latina	Somali	South Sudanese	Sudanese	Tamil	Punjabi
	(*n* = 35)	(*n* = 27)	(*n* = 17)	(*n* = 38)	(*n* = 11)	(*n* = 20)	(*n* = 12)	(*n* = 9)
Age range	19–50	18–70	28–46	19–68	30–45	26–54	26–50	25–45

Variable	*M* (SD)	*M* (SD)	*M* (SD)	*M* (SD)	*M* (SD)	*M* (SD)	*M* (SD)	*M* (SD)
Age	31.4 (9.1)	38.7 (12.5)	37.1 (5.6)	31.9 (10.4)	36.6 (6.2)	38.7 (7.5)	36.8 (7.3)	35.67 (6.22)
Years since migration	5.1 (4.1)	4.3 (2.1)	8.3 (4.9)	5.4 (3.1)	10.8 (2.1)	8.9 (3.4)	5.1 (2.5)	8.11 (2.37)
Number of children	3.3 (1.3)	2.7 (1.2)	1.5 (0.7)	3.7 (2.0)	4.5 (2.2)	2.9 (1.1)	2.1 (0.5)	2.40 (0.89)

	*n* (%)	*n* (%)	*n* (%)	*n* (%)	*n* (%)	*n* (%)	*n* (%)	*n* (%)
Have children								
Yes/pregnant	20 (57.1)	20 (74.1)	11 (73.3)	23 (60.5)	11 (100.0)	19 (95.0)	11 (91.7)	5 (55.6)
No	15 (42.9)	7 (25.9)	4 (26.7)	15 (39.5)	–	1 (5.0)	1 (8.3)	4 (44.4)
Religion								
Islamic	35 (100.0)	23 (85.2)	–	38 (100.0)	–	16 (80.0)	–	–
Christian	–	3 (11.1)	10 (58.8)	–	11 (100.0)	4 (20.0)	5 (41.7)	–
Buddhist	–	–	1 (5.9)	–	–	–	–	–
Hindu	–	–	–	–	–	–	7 (58.3)	5 (55.6)
Sikh	–	–	–	–	–	–	–	3 (33.3)
Non-practicing	–	1 (3.7)	6 (35.3)	–	–	–	–	1 (11.1)
Education								
Primary	7 (20.0)	6 (22.2)	–	8 (21.1)	3 (27.3)	4 (20.0)	–	1 (11.1)
Secondary	15 (42.9)	3 (11.1)	2 (11.8)	3 (7.9)	2 (18.2)	5 (25.0)	7 (58.3)	–
Tertiary	6 (17.1)	18 (66.7)	10 (58.8)	3 (7.9)	2 (18.2)	10 (50.0)	5 (41.7)	8 (88.9)
Nil	2 (5.7)	–	–	1 (2.6)	2 (18.2)	1 (5.0)	–	–
Other	1 (2.9)	–	–	2 (5.3)	2 (18.2)	–	–	–
No response	4 (11.4)	–	5 (29.4)	21 (55.3)	–	–	–	–
Relationship status								
Married/de facto	17 (48.6)	14 (51.9)	13 (76.5)	13 (34.2)	6 (54.5)	13 (65.0)	12 (100.0)	9 (100.0)
Single	12 (34.3)	7 (25.9)	2 (11.8)	14 (36.8)	1 (9.1)	1 (5.0)	–	–
Divorced/separated	2 (5.7)	5 (18.5)	2 (11.8)	8 (21.1)	4 (36.4)	6 (30.0)	–	–
Widowed	4 (11.4)	1 (3.7)	–	3 (7.9)	–	–	–	–

Australia and Canada were chosen as the geographical site for the research as they are similar geographically, economically and politically, and have comparable migrant and refugee populations. Our intention was to include adult women from a range of cultural backgrounds, religions, and age groups, who had migrated within the previous 10 years. The specific cultural groups were chosen through consultation between the research team and community stakeholders who partnered with us in the research. These stakeholders are involved with supporting or providing sexual and reproductive healthcare to migrant and refugee populations, and included migrant resource centers; sexual health, family planning and maternal health clinics; and community health centers, in both Sydney and Vancouver. The cultural groups selected were recognized as being underrepresented in previous sexual health research and were identified as underutilizing current sexual health services, despite reflecting a significant proportion of the recent migrant and refugee population of Australia and Canada. We excluded women from South-East Asia, as they have been the focus of a substantial proportion of sexual and reproductive health research in Canada and Australia, and women from China, who are part of very established communities in both countries, with established networks of health support.

### Procedure

Community stakeholders and key informants from the cultural backgrounds of the women we interviewed, including our community interviewers, were consulted in the development of the proposal, research design and methods, for guidance on the interview schedule and to gain cultural insight. These community stakeholders were identified through migrant resource centers that partnered with us in the research. Women were recruited to participate in the study through community support workers, community interviewers, pre-existing community groups, flyers, and snowball sampling. Before taking part, women were informed that participation would involve discussion of sexual and reproductive health. Any queries about participation were addressed verbally with a community worker in the first language of the participant to ensure understanding.

The interviews and focus groups were semi-structured and lasted an average of 90 min. Women were informed that the discussion would examine their experience of sexual and reproductive health. Consent was given by participants to audio-record all interviews and focus groups, with the exception of one interview where the participant declined to be recorded and extensive notes were taken instead. The interview and focus group schedule included open-ended questions exploring women’s experiences of menarche and menstruation, and their association with a sense of sexual embodiment; fertility and contraception; sexual rights and sexual agency (the right to say no to sex, sexual pleasure); sexual health screening; sexuality education; and sexual health information seeking. At the end of the interview, women were given information about sources of advice and information, if they wished to follow up issues discussed in the interview. The interviews were pilot tested prior to the research, in both a focus group and interview format (Ussher et al., 2012; Wray et al., 2014).

Individual interviews enable the collection of personal in-depth accounts, on a sensitive subject, that may not be disclosed in a group setting (Creswell, 2009). Focus groups provide a synergistic setting to gather insight into cultural and community norms, using interaction data resulting from discussion among participants (e.g., questioning one another, commenting on each other’s experiences) to increase the depth of the inquiry and unveil aspects of the phenomenon assumed to be otherwise less accessible (Creswell, 2009; Duggleby, 2005; Lambert & Loiselle, 2008). The combination of methods allows for examination of complementarity in analysis to enhance data richness: elaboration, illustration, and clarification across methods (Lambert & Loiselle, 2008). The focus groups were homogenous, as is recommended (Krueger, 2014), consisting of women from the same cultural group, but involved separate groups for married and single women where possible.

Using community interviewers is a methodology which has been successful in past sexual and reproductive health research with migrant women (Go et al., 2002; Morrow et al., 2008). The community interviewers were recruited specifically for this project, through partner organizations and other community networks. They were all migrant or refugee women, who had high school or tertiary education prior to migration. None had previous experience of qualitative research. Prior to commencing data collection, they received interview training in the schedule to be used in this research, in how to conduct conversational interviews, and how to transcribe data, by members of the research team, in a 1-day workshop. Ongoing support and advice was provided for the interviewers throughout the data collection process by a member of the research team, who commented on transcripts as they were submitted, gave advice on questions that were not fully addressed in the transcript, and supported the interviewers in the general data collection. Each community interviewer was responsible for conducting approximately five interviews and one or two focus groups. Interviews and focus groups took place at venues preferred by participants, primarily in residences or community facilities such as libraries and community centers and childcare was provided where necessary. The interviews and focus groups undertaken in English were conducted by AH, a member of the research team trained in qualitative research, who was not from a migrant or refugee background.

All women provided informed consent, and the research was approved by Western Sydney University and Family Planning University Ethics Committees and ethics committees of the project partners.

### Analysis

In our analysis, we adopted a material-discursive theoretical approach (Ussher, 1997a), embedded within a critical realist epistemology (Bhaskar, 1989). Described as a way forward for research examining health in a sociocultural context (Williams, 2003), critical realism is an epistemological standpoint which recognizes the materiality of the body, and other aspects of experience, but conceptualizes this materiality as always mediated by discourse, culture, and social or political practices (Bhaskar, 1989). This approach allows us to examine women’s experiences of sexual embodiment, the ways in which “our social and historical environments enter into and become entangled with our bodies” (Tolman et al., 2014, p. 761). This viewpoint emphasizes the ways in understanding how the sexual body is influenced by cultural norms and discourses, which constitute bodily feelings and behaviors (Bartky, 1990; Bordo, 1993), and which come to “constitute the phenomenology of embodiment” (Tolman et al., 2014, p. 761). We also drew on the concept of intersectionality (Crenshaw, 1991), an approach previously recommended for research on migrant and refugee women’s sexual health (Ngum Chi Watts et al., 2014; Salad et al., 2015). Intersectionality was originally proposed as a critique of feminist and anti-racism research that examined women’s identity through a lens of gender, or a lens of race, using an “either/or proposition” (Crenshaw, 1991, p. 1242), thus serving to marginalize women of color, whose experiences “are frequently the product of intersecting patterns of racism and sexism” (Crenshaw, 1991, p. 1243). More recent developments of intersectionality focus on the interaction and mutually constitutive nature of gender, race, religion, age, and other categories of difference in individual lives and social practices (Davis, 2008, p. 68). There is recognition that all people are characterized simultaneously by multiple and interconnected social categories; that a dimension of inequality or power is embedded within each category; and that the categories are properties of the individual in terms of their identity, as well as characteristics of social structures (Else-Quest & Hyde, 2016). Within this framework, categories such as gender and culture are recognized as complex and dynamic concepts, closely linked to the processes and structures embedded in society that shape individual and group behavior (Dyck, 2001).

**Fig. 1:**
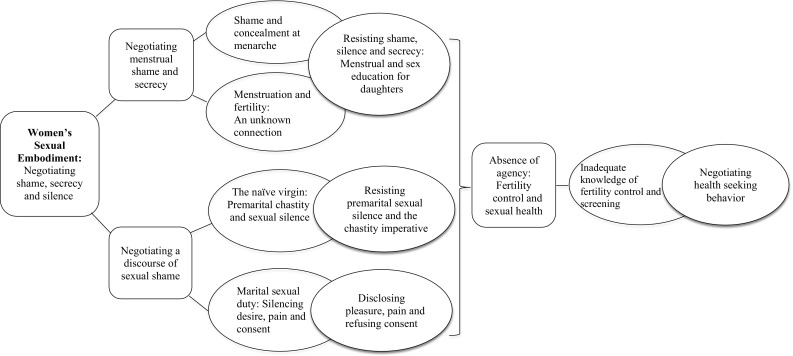
Thematic map

Thematic-decomposition was used to analyze the data (Parton, Ussher, & Perz, 2016; Stenner, 1993; Stenner, McFarquhar, & Bowling, 2011), a form of discourse analysis which attempts to separate a given text into coherent themes which reflect subject positions allocated to, or taken up by, a person (Davies & Harre, 1990). Analysis was inductive, whereby the development of themes was driven by the data and not by existing theory, research or hypothesis. Interviews and focus groups conducted in the first language of participants were transcribed and translated into English by the community interviewers who interviewed them. Interviews and focus groups conducted in English or with English spoken sections were professionally transcribed verbatim. Transcripts were integrity checked by listening to the audio recording and reading written text to ensure authenticity and accuracy. To improve readability, filler words such as “ah ha,” “um” and ‘mmm’ were removed. All participants were given pseudonyms. A subset of interviews and focus groups were read and re-read by three of the research team independently (JU, AH, and CM) to identify first-order concepts or codes, such as “menstrual learning,” “talking about sex,” “constructions of disease,” and “screening behaviors.” The coding frame was then discussed and modified by the whole research team, in order to ensure clarity, relevance, and appropriateness for addressing the research questions. The entire data set was coded by two members of the research team (AH and CM) utilizing the computer software NVivo, a program that helps facilitate the organization of coded data. Each researcher coded separate sections of the data independently, by extracting sections of text and filing them under the agreed coding frame. The accuracy of coding was assessed by a third member of the team (JU), who read through the coded data as it was progressing, and provided feedback. The codes were then grouped through a process of vigilant decision-making through discussion between JU, AH, JP, and MM, to create fewer, more distinct categories as the process continued. Following the coding process, each coded category was summarized in detail by AH and CM, to identify themes within codes, and participants who were reporting each theme, a process which served to highlight commonalities as well as unique stories across the data. This enabled us to identify how women discursively experience and construct their sexual and reproductive health needs in relation to their wider sociocultural contexts (Ussher & Perz, 2014) (see Fig. 1. Thematic map).

Throughout our analysis, we engaged in a process of reflexivity (which is germane to an intersectional approach), involving self-awareness of the intersubjective dynamics between the researcher and the researched, and critical reflection on how our social and cultural backgrounds, assumptions and positioning impacted upon the research process (Finlay & Gough, 2003). The research team consisted of white women, and women of color; women who had migrated and those born in Australia and Canada; academic researchers; and those working with migrant women in the community. Our different experiences were reflected upon in the design of the research, and the analysis and interpretation of data. We were also reflexive about the role of community interviewers, who shared many of the experiences of the women they interviewed, but also experienced differences in their views or experiences. This was discussed during the interview training, as part of encouraging interviewers to be non-judgmental, and was also something we considered in our analysis, and our suggestions for future research.

Within the quotes presented in this article “…” are used to identify sections of discussion not relevant to the analytical context. Further, “[]” signifies text that has been added by the authors to improve readability and meaning. Quotes are substantiated by the use of pseudonym, ethnic background, and age. Through our analysis, no notable difference was found between the accounts of women from Australia or Canada; therefore, no distinction is made with regard to current country of residence. No notable differences were found across individual interviews and focus groups; however, we have provided information of the type of interview conducted, as pseudonyms were not allocated to focus group participants. Where differences across culture, age or religions were identified, they are reported.

## Results

### Women’s Sexual Embodiment: Negotiating Shame, Secrecy, and Silence

Across all of the cultural groups, participants described shame as the dominant discursive construction of women’s sexual embodiment. This was associated with secrecy and silence, with public discussion of women’s sexual embodiment or experience described as culturally taboo. This shame, secrecy and silence had implications for women’s knowledge and behavior in relation to sexual embodiment and sexual health, potentially limiting agency and causing distress. While a discourse of sexual shame was adopted in many women’s accounts, there were also accounts of resistance, both before and after migration. Adoption and resistance of a discourse of sexual shame were not polarized and discrete subject positions, however. Women’s accounts demonstrated active *negotiation* of dominant cultural discursive constructions of women’s sexual embodiment in order to achieve a degree of sexual agency, yet also maintain cultural and religious identity. This is reflected in the overarching theme presented in this article “Women’s sexual embodiment: Negotiating shame, secrecy, and silence,” and the three sub-themes, examining the negotiation of menstrual shame and secrecy; negotiation of sexual shame; and absence of agency: fertility control and sexual health.

#### “Yesterday You Were a Girl, But Today You’re a Woman”: Negotiating Menstrual Shame and Secrecy

##### Shame and Concealment at Menarche

The construction of sexual embodiment as an object of shame, necessitating secrecy and silence, began with menarche, an embodied experience that signifies the transformation of a girl into a sexual woman. This is reflected in retrospective accounts of menarche signifying a girl’s ability to be married: “after I got my period I have to get married” (Sudanese focus group); “they tell you now you grow up. After period you can get married” (Hasina, Somali, age 25). For the majority of women, menstruation was positioned as “something dirty and something to feel bad about” (Latina focus group), or a sign of “dirt inside the body” (Sharifa, age 39, Iraqi), a signifier of abject femininity (Kristeva, 1982). Discussion of the process of menstrual bleeding was culturally “shameful”: “in Sudanese culture, it is shame to talk about it [menstruation]…They believe it’s very sensitive and private issue this why they don’t talk about it” (Saba, Sudanese, age 48); “everything [about periods] was so shameful, even we don’t have parents to talk to” (Erina, Somali, age 39). Talking about menstruation was described as “gossip” that women should “keep away from” (Darya, Afghani, age 24), or something that only “naughty girls” (Andrea, Tamil, age 26) would discuss. Breaking this taboo was “disrespectful” and a sign “you don’t have respect for yourself” (Suhaira, Afghani, age 20). The only exception to this positioning of menarche as shameful was found in accounts of a minority of women from Tamil and South Sudanese backgrounds, who described celebration of menarche through family rituals and practices, including ceremonies, parties and animal sacrifice. These rituals were described as more common in “older generations,” rarely practiced since migration, and not desired by young women, as they drew attention to menstruation: “it’s not good when people come over and say ‘oh, she got her period, now she’s a big girl’” (Suz, South Sudanese, age 42).

As a result of this culturally sanctioned secrecy and silence, many participants reported having had no knowledge of menstrual bleeding or its function prior to menarche: “I didn’t know what it was actually” (Tamil focus group), “I thought I was the only girl who got it” (Ara, Afghani, age 34). When recounting their menarcheal experiences, some participants thought menstrual blood was feces or urine: “I had no knowledge about it. I thought I had diarrhoea with blood” (Afghani focus group); “I thought I peed myself” (Hani, Somali, age 32). Others thought they had committed “a sin” or “done something wrong,” or that they had injured themselves, as illustrated in the accounts of two Afghani focus group participants: “I was thinking that I might have torn my vaginal area,” “I thought I must have ripped something in my belly.” As a result, many of our participants reported distress when they first bled: “I was really scared, I will never forget my experience” (Afghani focus group); “I couldn’t function like normally, I was very sad. I didn’t ask questions and people didn’t guide me” (Latina focus group).

Silencing and secrecy was also associated with reports of embarrassment at menarche, wherein adolescent girls reported being “ashamed,” as well as “shy” or “afraid” to disclose their first period, learning to conceal menstruation in isolation. Thus, Arliyo said “I hide it [period], I was hiding 1 year and they didn’t know it” (Somali, age 26), and an Afghani focus group participant told us “I remember that for about a year, not even my mum knew that it was happening, because in Afghanistan, we have, you know, a sense of shame.” Concealment of menstrual blood and self-surveillance was imperative: “I went to the toilet so many times…checking everything is ok”; “I was worried people could see blood spots on my clothes” (Afghani focus group). The imperative for concealment extended beyond menstrual blood to the menarcheal woman, with many participants describing practices of self-seclusion. This is illustrated in the following accounts: “I was so embarrassed I didn’t want to show my face to anyone…I locked myself in a room” (Hooria, Sudanese, age 35); “No I didn’t tell anyone I just put [on] pads and isolated myself. I can’t look to my father and mother faces” (Saba, Sudanese, age 48).

##### Menstruation and Fertility: An Unknown Connection

This culturally sanctioned secrecy and silence meant that many participants reported having received inadequate knowledge regarding the connection between menstruation, sexuality and fertility. As Arliyo (Somali, age 26) told us: “I didn’t know that, because nobody tells us, nobody talk about it. We don’t know how we get a baby.” Some participants entered into engagement or marriage with inadequate information, as Zinat’s account typified, “I was 19 years when I got married. When I got engaged, I experienced a lot of tension how to find out if one is going to have a baby. Till 19 years I did not know anything” (Punjabi, age 45). Some participants described learning the connection between menstruation, sexual intercourse, and pregnancy through sex education at school following migration: “I found out after a few years that my period and pregnancy were connected to each other when I came to Australia and I attended high school” (Fahmo, Somali, age 23). This illustrates the potential influence of sexuality education and migration on women’s sexual knowledge.

Some post-menarcheal girls did receive informal education about the association between menstruation and sexuality, primarily from sisters, aunts and friends. However, some participants were given inaccurate information, as the following account illustrates, “I was told that now I have started bleeding, and if I sit on my brother’s chair or maybe I wear his pants, I will become pregnant” (Somali focus group). Others were warned about contact with men, but didn’t understand why: “They say it’s not good when the boy touches you…I didn’t know the meaning of that” (Suz, South Sudanese, age 45). These beliefs served to regulate relationships between men and women, now that young women were capable of getting pregnant. As the South Sudanese focus group participant went on to say, “Once I got it [period], I was scared of men. Because my mum always told me these stories.” In contrast, young women who had received sexuality education through schools, parents or other family members, and knew about the association of menstruation with sexuality and fertility, positioned menarche as a welcome sign of normality: “I turned 16, exactly two days after, the period, it come to me, I was very happy, now was thinking I’m going to have kids because I have period” (Era, Sudanese, age 26). Such accounts were primarily from young women whose first menstrual period occurred after the majority of their peer group.

##### Resisting Shame, Silence, and Secrecy: Menstrual Education for Daughters

As all of our participants were adult women, accounts of shame, secrecy, and silence associated with menstruation were retrospective, illustrating the ways in which patriarchal family structures intersect with age, and culture. Very few women currently adhered to a discourse of menstrual shame. The majority of participants described resistance to the cultural taboo surrounding discussion of menstrual blood in order to prepare their daughters for menarche, as evidenced in the following accounts: “It’s very good to teach your daughters these things” (Homa, Afghani, age 40); “I treat my daughters not like what my mother treated me…I don’t want them to be shocked like I was” (Madina, Iraqi, age 45).

I want to avoid what happened to me when no one told me, so I told my daughter. I told her what was going to happen… So she already knows. Once she gets her period, I will know and my daughter knows (Sudanese focus group).

This negotiation of menstrual shame and secrecy resistance was not without difficulty, however, as many women expressed concerns about the process of providing menstrual education. For example, Asilah (Iraqi, age 34) said she would only tell her daughter about menarche when she asked her about it: “I wish she would ask me so I could answer her questions.” Ara (Afghani, age 34) gave a similar account, and relied on her daughter’s school to tell them: “If they hear and they come up to me and ask something, then obviously I will explain, but I’m sure in Australia once you go to school, you know about everything.” At the same time, a number of women had discussed menstruation with their daughters, but avoided explanation of the association of menstruation and sexuality or fertility: “We were raised just like, a little bit shy from those matters [sex]. Even I can’t talk with my daughter frankly” (Raana, Iraqi, age 43); “I told them it’s normal to have that changing process in a woman’s body. I bought them the things they need when they bleed….But I do not talk to them about sex” (Saafi, Somali, age 43). A minority of women gave accounts of warning their daughters to “watch their steps” (Amran, Somali, age 47), or “be careful, don’t mix with a lot of boys, just mix with the girls” (Sudanese focus group), reproducing the warnings they had received from their own mothers, without explaining the reasoning behind the warning. These accounts demonstrate the complexity of women’s negotiation of cultural discourse associated with menstruation, with certain cultural taboos more difficult to resist.

#### “A Lady Doesn’t Talk About These Things or Behave in That Way”: Negotiating a Discourse of Sexual Shame

The discursive association of sexual embodiment with shame, leading to secrecy and silence, extended to adult women’s sexuality. Talking about sex was positioned as shameful from both a cultural and religious point of view. For example, Hooria (Sudanese, age 35) told us that “it is shame to talk about sex with anyone in my culture and I feel embarrassed to talk about it” and Habibah (Iraqi, age 43) said “in religion it is not allowed to talk about our sexual relation with others.” Women who broke the taboo and talked about sex were positioned by participants as having “no values at all” (Akoi, South Sudanese, age 40); a “whore” (Ariana, Latina, age 40); “vulgar,” “not behaving like a lady,” or as a “woman with poor education” (Catalina, Latina, age 45); “not a good girl” (Eira, Sudanese, age 26).

##### The Naïve Virgin; Premarital Chastity and Sexual Silence

Cultural strictures surrounding discussion of sex were most severe for unmarried women and girls, illustrated in the following examples: “I can’t ask my sister because I am a virgin” (Hasina, Sudanese, age 25); “your mother would kill you if she heard that word [sex]” (Arliyo, Somali, age 26). A number of participants described sex education of their daughters as something they didn’t approve of: “I can’t accept it, because I came from this sort of cultural background, I feel I can’t accept it” (Jane, Tamil, age 32); “in Sudan, they think if boys and girls get any kind of knowledge about sex, they will turn into bad people…that’s why they don’t educate them” (Wafa, Sudanese, age 40).

This silencing and secrecy was attributed to the patriarchal cultural and religious dictate that unmarried women remain virgins, and thus “to even think about this [sex] is wrong” (Afghani focus group). Sex before marriage was described as “something very bad, something that it’s a sin, something that is not allowed, something that you don’t do” (Isabella, Latina, age 46), “forbidden” and “not lawful” (Hawa, Sudanese, age 30), and “sexual abuse for the woman” (Iraqi focus group). A number of participants described having felt “proud,” “pure,” “innocent,” “honorable,” and having “cleanliness in the heart” through being virgins on their wedding night—the antithesis of sexual shame. Conversely, women who broke the taboo by having sex before marriage were described as “disgraced” and needing to be saved by God to “show them the right path” (Afghani focus group), because of having been shamed. In some contexts, women were also at risk of violence, social ostracization, or were positioned as unmarriageable.

Taboos associated with discussion of sex pre-maritally meant that many participants had no prior knowledge about what sex involved before their wedding night, as these women told us: “I even couldn’t ask anybody because it was something forbidden” (Wafa, Sudanese, age 40); “I didn’t know how you even [do] sex” (Suz, South Sudanese, age 42) when they got married. This lack of knowledge was associated with difficult first sexual experiences, as demonstrated by the following accounts, “It was a complete surprise to me if I am honest with you…I was really scared” (Banoo, Afghani, age 28); “the first day for my wedding day I thought he is doing the wrong things, and I start screaming” (Nasira, Iraqi, age 52); “after the first night, I felt like running back home to my mother” (Anju, Tamil, age 44). Reports of unexpected sexual pain on the first occasion were very common: “I felt that it hurt and I got a bit upset. I couldn’t allow him to do anything, and he was a bit disappointed too” (Aameeka, Tamil, age 40); “I didn’t enjoy it because it was very painful, very, very painful. And, I was like, ‘Oh my God, what is this’?” (Isabella, Latina, age 46). For some women, their surprise was that sex was pleasurable or “okay”: “I didn’t [know anything] but all went okay…he was gentle and it didn’t go for long” (Sumi, Tamil, age 37); “it was very, very good. And after, I told him, ‘I had a good experience’” (Laughs) (Saadia, Iraqi, age 30). Positive experiences of first sex were in the minority, however, with the majority of women reporting fear, embarrassment, discomfort or pain.

##### Resisting Premarital Sexual Silence and the Chastity Imperative

Adherence to premarital sexual secrecy and silence was not universal. A number of women gave accounts of resistance through seeking out sexual information before they were married, as Nasira (Iraqi, age 52) told us:

My mum and auntie, they don’t allow us to read this book because it’s about sex, it’s about people asking the questions. In our age, they say it’s not allowed, so when we buy it, we hide it. We read it and hide it, this book.

Other women talked about seeking out information on the internet, or talking about sex with close friends. As Akoi (South Sudanese, age 40) told us: “I had a friend and my friend had another friend and we actually talked about sex…it’s part of life that’s how we actually talked about it.” Many women positioned sex education of their daughters as important, such as Fahmo (Somali, age 23) who said, “I just think that sex education is very important for young girls as well as learning about periods.” The acceptability of sex education in Australia and Canada contributed to this open communication, allowing women to let go of a sense of shame: “before I was thinking shame, I’m not feeling shame now. I answer them everything they are asking me…I feel more freedom. Before, there was something I’m hiding, I’m not hiding now” (Erina, Somali, age 39). Several women were interested in finding out ways to approach talking about sex with their daughters as the following accounts highlight: “I would actually want to arm myself with all the right information so I could actually educate my daughter how she can go about those things” (Akeck, South Sudanese, age 31); “since I am a mother, I would like to have information about sexual education for kids and young people. I would like to have some advice or guidance on how to approach these kind of issues with my daughter” (Mariana, Latina, age 38). One woman learnt about sex, as well as how to educate her children, through attending her child’s school sex education class, described as “me and my child learning at the same time” (Tamil focus group).

A number of women resisted a discourse of sexual shame through challenging the chastity imperative. Some participants said that sex before marriage could be acceptable if you were intending to marry the man, or that it should be a personal choice. For example, Joy (South Sudanese, age 45) said that “if it happens, it happens” when asked about her views on her daughter having premarital sex. The chastity imperative was also resisted through questioning of the sexual double standard wherein men could have sexual experience before marriage and women couldn’t: “The Somali men, although they are not virgins, they like to have a virgin wife and that is not fair” (Somali focus group). A small minority, most commonly from Punjabi and Latina backgrounds, acknowledged that they had engaged in sexual intercourse before marriage: “I think that live-in relationships are a very good test to have before marriage, maybe a little more practical, I think than the way we go about things traditionally (Anu, Punjabi, age 35):

I was a rebel…I had sex before marriage….I personally believe that no women should marry being a virgin, right? You cannot get married to someone without knowing how you are going to work together in bed. You don’t know if you are sexually compatible (Sofia, Latina, age 40).

Women’s resistance to the chastity imperative was facilitated by secrecy and silence: “no-one knows, it’s between you and your partner” (Afghani focus group); “that’s up to you and god” (Erina, Somali, age 39); “It’s something you have to keep secret because it’s still taboo” (Afghani focus group); “I’m sure lots they have it [sex before marriage], but in a secret way” (Raana, Iraqi, age 43); “if it happens in our society, no one will ever talk about it” (Wafa, Sudanese, age 40). This is evidence of silence and secrecy serving as a means of resistance, described in previous research as a “negotiated silence” (Kebede et al., 2014, p. 673). Secrecy could also be maintained through hymenoplasty, allowing women to engage in premarital sex, yet appear to be a virgin on their wedding night: “there is some doctors they do repair of hymen” (Nadiya, Iraqi, age 70,); “one of my classmates in high-school, she was mentioning that she was thinking of doing a hymen repair” (Ariana, Latina, age 40).

Silencing and secrecy extended to women’s self-disclosure within the interviews, as premarital sex was primarily discussed in relation to ‘other’ women. Those specifically mentioned were women of a “lower social class,” women “from the country,” or “recent generations,” as a Sudanese focus group participant commented: “these types of relations [exist] and happen and especially nowadays, especially the recent generations…But my religion prevents me from doing these things.” This suggests that women could negotiate a discourse of sexual shame by discussing premarital sex existence without judgment, yet at the same time maintain their own position as “good women.”

##### Marital sexual Duty: Silencing Sexual Desire and Pain

While premarital sex was prohibited for women, marital sex was imperative, described as a “duty” and “the meaning of marriage,” within religious and cultural discourse. For many women, marital sex was associated with silence, as sex was a forbidden topic between a woman and her husband, as Arifa (Iraqi, age 48) explained:

The culture does not permit such conversation…We are not allowed to talk about sex, and some sexual conversations we are not allowed to talk about, not even in our bedroom… there are some issues we can never talk about, it’s a taboo.

Lack of sexual communication and knowledge meant that some married women had no concept of their own sexual pleasure. Anu (Punjabi, age 35) explained, “so actually we don’t talk about sex at all, so I have no idea whether we’re meant to enjoy it or not to be honest.” A Latina focus group participant explained that an “open” environment with greater sexual communication and education would have enabled her to enjoy sex, but because sexual knowledge was taboo she experienced feelings of “guilt” and “shame.”

For many participants, women’s sexuality was constructed as something that is to give pleasure to a man, rather than for themselves, “to give joy to her husband and to bear children,” in the words of Nasima (Iraqi, age 43). She continued: “it is rare to find a man who cares about giving his wife pleasure. Beside some women do not know any better and are very naïve.” Self-pleasure through masturbation was something the majority of women had no knowledge about, or were judgmental of, saying “I never knew this type of stuff exists,” “it’s dirty,” or “such a bad thing to do” (Afghani focus group). Heterosexism, in its association with culture, was clearly apparent as same-sex relationships were also something many participants had not heard about before moving to Australia or Canada, described as something that didn’t exist outside of Western culture: “It’s not in our culture, it’s not in our religion, it’s not something we chose” (Somali focus group); “we don’t have this” (Amer, Sudanese, age 34); “in my opinion, it is religiously illegal ‘haram’ [sinful] and for my opinion it is completely totally refused” (Iraqi focus group). If same-sex relationships were acknowledged, they were positioned as “secret”: “these relations are found in our societies but in secret because it is forbidden” (Iraqi focus group). Only a small number of women, primarily those educated in Australia or Canada, resisted condemnation of same-sex relationships. Some adopted a discourse of sexual freedom and choice: “with the children that have grown up in the western culture where there is a freedom of choice, so you can do whatever you like for yourself” (Akon, Sudanese, age 30). Others adopted a discourse of sexual rights, as Samira (Afghani, age 21) commented, “I personally think that so long as people don’t harm you, then you should not interfere with their lives.”

Some of the women we interviewed did provide accounts of sexual pleasure and desire, as Arifa (Iraqi, age 48) told us”: I get the feeling that I need a man in my life, it’s hormones, sometimes I strongly feel I need a man, and want to have sex.” For some women this was a cultural and religious imperative, as Samira told us: “In Islam, you are supposed to…You’re supposed to enjoy it [sex]” (Afghani, age 21), and “if he can’t please her, then he has to actually answer for it on judgment day” (Homa, Afghani, age 40). However, if women adhered to the cultural prohibitions surrounding sexual communication, they were not able to initiate or express their desire for sex with their husband. For example, one participant said, “[t]his is a culture thing. So it’s an understanding in the culture. Women never ask for sex” (Sudanese focus group). Another participant said, “[a]s a woman, in our culture, we do not say, and just, however, badly we really want to have sex that night, it’s not just easy to say, we just don’t, we don’t even talk about it” (Somali focus group). For a number of women, initiating or desiring sex felt wrong religiously, as an Afghani focus group participant commented, “I felt a sense as if I’m committing a sin, it felt sinful.” She went on to say, “even though I knew he is legally my husband and it’s okay, it’s allowed…it felt very strange and uncomfortable if he didn’t initiate it. I just, I wouldn’t.” Women who initiated sex or expressed their desires were at risk of being negatively labeled and stigmatized within a discourse of sexual shame: “everybody sees that kind of woman as a whore, as somebody that doesn’t have values or principles, and not a good education. They perceive her as a bad woman” (Sofia, Latina, age 40); “it’s not acceptable, it’s shame… they never show their desires to their husbands” (Husna, Sudanese, age 45).

Silence, secrecy and shame extended to experiences of sexual pain, a common occurrence for many women, as Darya told us “it hurts every time” (Afghani, age 24). Some participants endured painful sex because of cultural norms to remain silent, as Hooria (Sudanese, age 35) said:

I feel pain in my vagina and my body[is] sore and can’t move especially the hours after the sex…I can’t talk to my husband or complain…I have to keep quiet. In my culture its shame to talk about this pain, it is considered a normal [part] of having sex.

In Hooria’s account, painful sex was normalized, associated with vaginal tightening, and repair of a previously infibulated labia, after childbirth, “to be as virgin and to make the husband happy and enjoying sex.” Other women associated pain with sexual frequency, but would not tell their husband of their discomfort as this would be “annoying” or he “would not understand,” as Arifa said: “he considers it joyful, but I consider it as painful especially if it’s few times a day, it becomes annoying too. He would not understand, so why would I tell him. It will not make a difference” (Iraqi, age 48). Some women also spoke of being “scared” (Shiwa, Afghani, age 50) of violent repercussions if they discussed sexual pain, which could imply refusal of sex, as Akoi said of her relationship with her ex-husband: “we just want him to get and do it and finish and then sleep, that’s all. And if you don’t, if you say no, you will be beaten” (South Sudanese, age 40).

##### Sexual Consent: “A Woman Is a Man’s Property”

Silence extended to the issue of sexual consent. Many participants gave accounts of having no consensual rights in relation to sex with their husbands, feeling unable to refuse sexual advances, as Akoi (South Sudanese, age 40) explained, it was considered a man’s “right” to have sex because “they pay dowry, so you are owned…like she is your property.” Arifa (Iraqi, age 48) said “It’s not my right to say no.” For a number of participants, refusing to have sex was considered a “sin” (Afghani focus group). As Amran explained, “even if you don’t feel like doing it, you just say, “yes,” because you’re scared of being cursed by the angels” (Somali, age 47). Other participants positioned having sex a wife’s duty out of “respect” for their husband, or “respect for his desire” (Kamelah, Sudanese, age 36). For example, Saba (Sudanese, age 48) stated:

Even if I am not happy I can’t talk at all, women has to have sex, including me, when the husband asks for it, even if women have no desire to have it. They have to respect the husband’s desire.

There was also a risk of their husband leaving if a woman said no to sex, as a Somali focus group participant told us “if you don’t want to have sex and it bothers you, allow him to marry another woman…But if you want to have him, just make sure that you are ready whatever time he wants.” If a woman did attempt to say no, it was deemed unacceptable by many husbands. For example, Faasio (age 32, Somali) described her husband as “cranky and angry” if she tried to refuse sex.

##### Disclosing Pleasure, Pain and Refusing Consent: Negotiating Sexual Shame Within Marriage

Negotiation of a discourse of sexual shame within marriage was evident in accounts of resistance to the cultural dictate that married women never discuss sex, or refuse sex, with their husband. A number of women described marital relationships where open communication about sexuality had always been the norm, as one Iraqi focus group participant commented “we always talk about everything [to do with sex].” A Latina interviewee, Mariana (age 38) described openness about sexual desire and sexual pain across the course of her relationship:

I would feel free to ask for certain things and to communicate my needs and pleasures, the way that I like to be touched, and all that. And the same when, sex started to become painful after I got my baby, I don’t have much lubrication these days, and the first time we had sex, after that, it was extremely painful. And yes, I was able to tell him, let us stop because this is being like a torture for me.

A small number of participants told us that since migration they saw sexual pleasure as their right, “…of course your society is opened so we learnt a lot from your society so I see that it’s my right to tell him what I enjoy and what I don’t enjoy in the sexual relation” (Sudanese focus group). Saadia (Iraqi, age 30) said that it was now “easy [to] talk about the positions you want” when having sex “to have different positions.” Several participants told they were now able to say no to sex, because they “have rights about what their choices are and they actually communicate that” (Akoi, South Sudanese, age 40).

When I came to Australia we go to school and learn the right of the woman…Women when she say’s no, means no, you can’t force her…so that’s why I say we get peace in Australia because we are independent now, we do our own thing by our self, no beating up…no someone force me all the time to have sex, whether you’re sick, whether you don’t want it (Suz, South Sudanese, age 42).

Others said no to sex, but felt guilty: “more often than not, I do say no. But I do feel that it hurts him more than anything else” (Anu, Punjabi, age 34). Other women resisted the marital sex imperative by positioning unwanted sex as ‘rape’. For example, Najiba said “[e]very night he used to rape me and that affected my health” (Afghani, age 64), when describing her relationship with her ex-husband. In this vein, some women saw a need for the men in their community to be educated about sexual health “because their information about sexual health is not enough” (Husna, Sudanese, age 45). For example, Saba (Sudanese, age 48) said:

The focus has to be on men in regards to sexual health education because their information and knowledge about sexual health is very poor. They want to apply the way they grew up in this country. They have to understand that forcing women for sex is rape and it is a crime in Australia.

These accounts are evidence of women’s adoption of a human rights-based sexual health discourse, in order to attain sexual agency, since migration, as reported in previous research (Quelopana & Alcalde, 2014).

#### Absence of Agency: Fertility Control and Sexual Health

##### Inadequate Knowledge of Fertility Control and Sexual Screening

Cultural and religious taboos associated with women’s sexual embodiment had broader implications for their knowledge and behavior in relation to sexual health. Fertility control through contraceptive use was subject to silence and secrecy for many women. For example, participants told us “I haven’t talked about it to anyone…Yea, our friends are very secretive, they don’t talk about, yeah, I don’t even talk that topic” (Aameeka, Tamil, age 40); “no, I don’t know anything about it [contraception]” (Amaal, Somali, age 42); “I have no idea about contraception” (Akeck, South Sudanese, age 31). A number of women told us that contraception was forbidden within their religion, as women were expected to bear children, suggesting that contraception use would be associated with shame: “To reject having a baby is forbidden in Islam” (Suhaira, Afghani, age 20); “no the religion does not allow woman to use contraceptives” (Hido, Somali, age 68). Others said that they needed permission from their husband: “I think the men should decide…in our culture every decision…you have to get permission from the male” (Safia, Afghani, age 28); “the lady have to tell the man” (Joy, South Sudanese, age 45). A woman who used contraception without telling her husband was at risk of violence or abandonment, so she had to keep it secret: “I use it, but still men don’t like it…I used to hide them from him sometimes…we had a fight and he hit me” (Faaiso, Somali, age 32); “if you make the medicine to stop babies and the man says no…he may separate with the wife…and will marry a new wife” (Elmera, Sudanese, age 34).

Some women did receive information about contraception from family or friends, from school, the family doctor, women’s health groups, the media, or within a refugee camp, and a number of women were using IUDs, the contraceptive pill, implants, or natural methods. However, incorrect knowledge about contraception was common. Participants told us they believed that contraception would impact negatively on their reproductive function, causing “a blockage,” making a woman “bleed to hell” (Akoi, South Sudanese, age 40), or that “taking a pill every day will get to your blood” (Afghani focus group), which “may damage your next baby…it may affect the next generation” (Safia, Afghani, age 28). Contraception before the first child was born was considered to be particularly damaging:

[Contraception is] not allowed at the beginning. You can’t take it before you have a baby. They even put in our minds that if you get contraception before they first come in and see you, you will never get pregnant anymore (Iraqi focus group).

Others reported more generalized health consequences, saying it “affects your organs” (Arliyo, Somali, age 28), “can give you some kind of sickness” (Lokoya, South Sudanese, age 42), or that “it creates a whole lot of other damages” (Afghani focus group). For these reasons, many of women we interviewed said they “don’t think of using it.” One of the consequences of poor or incorrect knowledge was unwanted pregnancy. For example, an Afghani focus group participant told us, “two of my children are a mistake,” and Asilah (Iraqi, age 34) said “the first baby I was so happy, but I surprised when I was expecting the second one. I don’t know how to control [having babies].” For other women, unwanted pregnancy was followed by abortion: “I got a termination two times” (Tamil focus group); “I fell pregnant 5 times; I did abortion because I didn’t want more children” (Najiba, Iraqi, age 64). While abortion was described as “as sin” or “illegal,” it was justified by the family being poor and “want[ing] finance” (Andrea, Tamil, age 26), or a woman being pregnant before marriage. It was usually conducted “before the fetus is old and it becomes illegal and wrong” (Habibah, Iraqi, age 43) and in secret: “never share with family members” (Andrea, Tamil, age 26). However, unwanted pregnancy and abortion could also function as a motivation for women to seek contraceptive information, as Ariana (Latina, age 40) commented “I thought that this cannot happen to me again. I cannot get pregnant again, so that was a turning point in my life for me to start to get more informed and more proactive around contraceptive methods.”

Absence of open discussion about sexual health also meant that some participants knew little about the existence or purpose of cervical screening. For example, a focus group participant asked, “do we need to do it still? Is it important?” The participant went on to say: “Isn’t it lack of knowledge? Lack of knowledge is the main thing…we don’t know what it is, what is that, how much is that important for us and things like that” (Tamil focus group). Similarly, Akeck (South Sudanese, age 31) stated about her lack of pap smear knowledge, “Yeah, I don’t know anything about them, it’s very shocking.” Equally, few participants knew about the existence or function of the human papillomavirus (HPV) vaccine, even though many women had teenage girls who are the target of preventative health programs. While a number of women told us that they “don’t know about it,” others did not see its relevance for their daughters: “if my daughter said that she has no sexual relations, why I give her this vaccine?” (Iraqi focus group). Others held misconceptions about the vaccine: “I think maybe the injection itself is causing cancer” (Somali focus group).

STIs were also a source of confusion for some women, or positioned as not relevant “because we don’t have sex with other men” (Tamil focus group), or “I don’t know much about that” (Banoo, Afghani, age 28). This lack of knowledge was positioned as a cultural norm, maintained by silence: “in my culture, we don’t know anything about that…I’ve never come across [it], no,” said Jane (Tamil, age 32). Others said that they only knew about HIV infection, or knew friends or relations that had experienced a STI, including urinary tract infections, or vaginal infections; however, women had little knowledge about what this entailed, or how to recognize symptoms. Several participants, most commonly from the Somali community, told us that they preferred not to have any discussion or knowledge about STIs due to constructing disease in a fatalistic way:

No, I don’t think about these things, you know, whatever will happen to me, it has already been decided by God. If things will happen to me, it will happen to me no matter what, and if it doesn’t happen, it doesn’t happen (Amran, Somali, age 47).

Similar accounts were found in relation to discussion of cervical cancer screening: “if it is meant to end your life then it will, nothing can prevent that” (Hoodo, age 29, Somali); “if anything happens, it happens” (Jane, Tamil, age 32). This suggests that the intersection of religion and culture, associated with cultural constructions of illness as determined by a god or fate, may serve to reinforce silence and secrecy associated with women’s sexual embodiment.

##### Negotiating Health Seeking Behavior

As a consequence of the silence, secrecy and shame surrounding women’s sexual embodiment, a number of participants told us they were reluctant to seek professional medical advice for reproductive and sexual concerns. Some women were reluctant to “expose the body” (Andrea, Tamil, age 26) to health care professionals. A Somali focus group participant explained that during the delivery of her baby, “I kind of let this male doctor to attend to me, but on the condition that when I’m having the baby, when I’m delivering the baby, he should not look at me. I mean, he should not look at my vagina.” Seeking help often occurred late and only in extreme cases, as Arliyo (Somali, age 26) said, “they just go to doctors when they are just dying.” In this vein, a Latina focus group participant went to the doctor after over 20 years of experiencing extreme menstrual bleeding:

It was very traumatic because I used to bleed a lot, like a lot…like so much, I would have to change the feminine paper every two hours and experienced like that for about 20, 24 years, until my mum brought me to the doctor because it was not normal. I would bleed so much that, to the point that I will faint, so I would faint almost every month, every time that I will get my period.

This delayed action left the participant feeling traumatized, “I couldn’t really function like normally…basically I was very sad. I didn’t ask questions and people didn’t guide me.”

In another example, Hawa (Sudanese, age 30) preferred natural methods and self-treatment due to the shame of discussing menstruation with a health care professional. She said she would “maybe” see a doctor “if bleeding stops completely” because “in our culture, it’s, it’s shameful to actually go to the doctor and talk about those things…I would eat fruits or try to help myself before going to the doctor.” Other participants chose not to seek medical help. For example, Akeck (South Sudanese, age 31) refused to go to a post-caesarean check-up even though she had been bleeding for an extended time:

I had my daughter, I had a total caesarean, and like the bleeding actually did not stop for certain times, but I did not actually go to see a doctor…I think it was six months after that…But…I failed to see my GP.

In the context of experiencing painful sex, Saba (Sudanese, age 48) “kept quiet and didn’t tell anyone” and Mariana (Latina, age 48) had not “taken any action or been proactive.”

Conversely, many women described a greater openness about their own sexual health concerns since migration, as evidenced by the following accounts: “talking about sexual health is very important. Thankfully, we are here in Australia so we can talk about it openly. Because back home, we can’t talk about it” (Sudanese focus group); “here I feel really comfortable about obtaining any kind of information related to my sexual health, but in Sudan and Saudi Arabia, I feel embarrassed to ask for this kind of information” (Wafa, Sudanese, age 40). This openness facilitated women obtaining knowledge about sexuality and sexual health, as well as seeking professional support if they experienced difficulties: “I have gone to the doctor about sex because my libido is quite low” (Anu, Punjabi, age 35). Some women dealt with their own feelings of embarrassment or shame by seeking help in educating their daughters, illustrated in the comments of a Sudanese focus group participant:

Before I told my daughter, I was scared to tell her about the period. Because my daughter might misunderstand me. I was scared and shy to talk about this topic. But what happened is I went to a migrant resource centre and there was a lady talking about women’s health. She talked about the periods and how to tell their daughters. I learned from that session I had to tell my daughter and I found it easy to handle.

A number of participants reported having changed their sexual and reproductive health screening behaviors post migration, for example, Hooria (Sudanese, age 35) told us that she now has regular pap smears, “I wasn’t aware of it before I came to Australia, my family doctor told me about it, I got all the information and I do it on regular basis.” A Tamil focus group participant told us that since migration they now “talk more” about contraception, although on arrival they had “nil” contraceptive knowledge. Akeck (South Sudanese, age 31) sought information about contraception. She countered the reproductive imperative discourse by saying, “information that educating young people, how they can protect themselves, just to enjoy or play, just not to have babies when they are not prepared to have babies, it is a critical, very critical thing that is very important.” Other women talked of seeking contraceptive advise from “older women in the community” (Afghani focus group), and of having positive experiences of fertility control, as Nasira (Iraqi, age 52) said of her use of an IUD, “it was really good.” However, many women wanted more sexual and reproductive health information, such as Setara (Afghani, age 23) who said, “I think in regards to different diseases, sexually transmitted diseases I think, I usually get scared about them, I want to know about them.” Other women wanted information about contraception, sexual pain, breast cancer, cervical screening, and menopause, suggesting there is a need for culturally safe sexual health information, in order to improve women’s sexual and reproductive health.

## Discussion

The sexual embodiment of migrant and refugee women is constructed and experienced in the context of transnational migration and the ensuing configuration of identity, wherein women negotiate diverse discourses, and patriarchal heterosexist constraints as mediated through culture (Sargent, 2006). Across each of the cultural groups who took part in this study, traditional cultural and religious discourse positioned the ideal woman as silent in relation to sexual embodiment, lacking in sexual knowledge and experience prior to marriage, and passive and receptive in relation to heterosexual marital sex. Women reproduced and resisted a discourse of sexual shame in order to account for, and make sense of, their sexual embodiment and sexual health. These findings confirm the findings of previous qualitative research with migrant and refugee women, that identified shame associated with menarche and menstruation (Cooper & Koch, 2007; Sommer et al., 2015), premarital sex (Kebede et al., 2014; Meldrum et al., 2014), discussion of sexuality (Dean et al., 2017; Quelopana & Alcalde, 2014), STIs (McMichael & Gifford, 2009), HPV inoculation and cervical cancer screening (Salad et al., 2015) and contraception use (Ngum Chi Watts et al., 2014; Sargent, 2006). This is a matter of concern for those interested in migrant and refugee women’s sexual health and well-being, as sexual shame is associated with absence of knowledge and communication about sexuality, manifested in secrecy and silence, and has an impact on sexual health and health seeking behavior, as evidenced by women’s accounts in the present study.

In addition to lack of preparation for menarche (Uskul, 2004), menstrual shame has been linked to increased sexual risk-taking (Schooler, Ward, Merriwether, & Caruthers, 2005) and embarrassment toward other reproductive functions, such as breastfeeding (Bramwell, 2001; Johnston-Robledo, Sheffield, Voigt, & Wilcox-Constantine, 2007) and childbirth (Moloney, 2010). Shame associated with premarital sexual knowledge or activity prohibits single women from accessing sexual health information or services (Beck et al., 2005; McMullin et al., 2005; Meldrum et al., 2014; Wray et al., 2014), with personal reputation or family honor jeopardized if it is known they are engaging in premarital sex (Quelopana & Alcalde, 2014; Rawson & Liamputtong, 2010; Ussher et al., 2012). The positioning of discussion of sexuality as disrespectful, indecent and culturally inappropriate results in sexual health concerns not being addressed with partners, family members, or health providers (Kebede et al., 2014; Quelopana & Alcalde, 2014; Rawson & Liamputtong, 2010; Rogers & Earnest, 2015). Inadequate knowledge about sexual desire, satisfaction or pain can lead to distress, isolation and underutilization of proactive coping strategies (Ussher et al., 2012; World Health Organisation, 2009), and an “absent discourse of desire” (Tolman, Impett, Tracy, & Michael, 2006), with implications for women’s sexual subjectivity (Fine & McClelland, 2006; Ussher, 1997b). Cultural constructions of the etiology of illness, or the efficacy of interventions, may inhibit access to sexual health services (Gagnon et al., 2002; Manderson & Allotey, 2003; Salad et al., 2015), with sexual and reproductive health services seen as culturally inappropriate (Guerin et al., 2006; Richters & Khoei, 2008), threatening fertility or the virginity imperative (Ngum Chi Watts et al., 2014; Salad et al., 2015), or undermining patriarchal power (Sargent, 2006). This has implications for the ability of health services providers to address migrant and refugee women’s sexual health needs, with implications for sexual health, pleasure and well-being.

The discourse of sexual shame is located in patriarchal and heterosexist cultural and religious ideologies, legitimating conjugal, familial and institutional regulation of women’s sexuality. Internalization of sexual shame, through a process of subjectification (Foucault, 1979), leads to women engaging in self-surveillance and self-policing: not speaking of their sexual embodiment, or their sexual needs and concerns (Hawkey, Ussher, Perz, & Metusela, 2016b). This is reinforced by fear of the material consequences of transgression, which include stigmatization, social exclusion and violence, as illustrated by the accounts of participants in the present study. However, women are not simply positioned within existing discourses, but can re-position themselves, variably adopting, resisting, negotiating and tailoring discourses associated with sexuality to achieve a desired sexual subjectivity (Day et al., 2010). In accounts of negotiation women reproduced *and* resisted a discourse of sexual shame. As Brown (2007) has argued, the “both/and” position “honors women’s agency and power while not minimizing the impact of oppressive social discourses and social relations” (p. 275). “Both/and” negotiation allows acknowledgement of the material consequences of transgression of a discourse of sexual shame *and* women’s agency and power in negotiating a degree of sexual agency. From this perspective, reproduction and resistance of discourse overlap rather than being discrete and separate processes (Day et al., 2010).

In the present study, many women adopted a human rights-based sexual health discourse in order to resist traditional cultural and religious constructions of sexual embodiment, allowing them to communicate or gain knowledge about sexuality and develop sexual agency, or to allow their daughters to do so. Participants negotiated a discourse of menstrual shame through acknowledging the cultural positioning of menstrual blood as abject, at the same time as contravening practices of secrecy and silence in order to educate their daughters, or to critically reflect upon their own menarcheal experience (Hawkey, Ussher, Perz, & Metusela, 2016a). As reported previously (Cooper & Koch, 2007; Kissling, 1996), those with little menarcheal support themselves were those who intended to provide their daughters with menstrual education. Negotiation of a discourse of sexual shame was evident in accounts of women seeking information about sexuality, and in non-judgmental accounts of “other” women’s pre-marital sexual practices. As premarital chastity is associated with a woman’s self-worth and self-respect (Salad et al., 2015), and open transgression with cultural and religious sanctions, it is not surprising that few women adopted a position of open resistance in this sphere, through admitting to pre-marital sexual experiences. Further negotiation of a discourse of sexual shame was evident in accounts of women discussing sexual issues within their marriage, refusing marital sex, discussing contraception, controlling their fertility, and accessing sexual health services. These accounts give us insight into the shifting balance of power between women and men in migrant and refugee communities (Fábos, 2001) and the negotiation of women’s sexual embodiment in the context of intimate relationships.

A number of intersecting factors facilitated women’s ability to negotiate traditional discourses associated with feminine sexuality. Migration and a process of acculturation provided alternative discourses to conceptualize sexual health, a stronger legal framework that facilitated resistance to sexual violence, and access to sexual health services that promote agency in relation to fertility control (Rogers & Earnest, 2015; Sargent, 2006). Generational changes within cultural groups, as well as social class, and rural/urban influences, were also influential factors, as reported in previous research (Fábos, 2001), as were the particularities of a woman’s relationship with her family, and more specifically with her husband. There were also some differences across cultural and religious groups, in terms of menstrual celebration being practiced by Tamil, Sudanese and South Sudanese women, premarital sex being more openly negotiated by Punjabi and Latina women, and women’s sexual pleasure as a religious right only acknowledged by Islamic women. While it is important to recognize and acknowledge the commonalities across cultural groups in terms of their negotiation of a discourse of sexual shame, it is also important to acknowledge differences. It is the intersection of the social categories of gender, age, culture, religion, and marital status, resulting in a “crossroads of multiple oppressions” (Marecek, 2016, p. 178), that influences migrant and refugee women’s construction and experience of their sexual embodiment. This suggests that an intersectional theoretical framework (Hankivsky & Cormier, 2009) is essential for future research in this field, and that structural inequalities and imbalances of power which influence women’s sexual and reproductive health must be acknowledged and addressed. In providing culturally safe services and support for migrant and refugee women’s sexual health, it is important for health educators and service providers to understand and acknowledge the cultural and religious frame of reference within which women construct and experience their sexual embodiment (Keygnaert et al., 2014). However, it is also important to acknowledge the fluid and dynamic nature of differences between women, the meaning and experience of simultaneously belonging to multiple intertwined social categories (Else-Quest & Hyde, 2016), acknowledged within an intersectional approach (Ngum Chi Watts et al., 2014; Salad et al., 2015), and women’s capacity to negotiate competing discursive constructions of sexuality and sexual embodiment.

There are a number of strengths and limitations of this study. Strengths include the majority of participants being interviewed in their first language, which allowed for experiences to be explored in-depth without limitation of language, and participation of women who were recent migrants and might not be fluent in English. The use of an English-speaking interviewer for the remaining proportion facilitated examination of whether accounts were being modified in the presence of a community interviewer, something we did not find. Interviewing a relatively large number of women from diverse cultural groups made both within and between cultural group comparisons possible. The use of both focus groups and individual interviews was a strength, as it allowed for in-depth discussion of individual women’s experiences. The fact that we didn’t find notable differences across the interview modalities was unexpected. What was notable was that many women were reluctant to discuss sexual matters in depth, regardless of the interview modality, and many of the community interviewers were reluctant to pursue the matter. This may reflect cultural restrictions on discussion of sexuality, reflected in our analysis. The fact that our interview schedule started with the discussion of menstruation, a topic all women were comfortable discussing with interviewers, may have facilitated discussion of sexuality later in the interviews, as women had gained a sense of comfort and confidence in the interview process. Limitations include the fact that researchers could not back-check translated transcripts for accuracy due to limitations in resources; participants were a convenience sample; women were retrospectively reflecting on some of their experiences; and women’s stories shared in this research might not necessarily be representative of their culture of origin, given we spoke to a small subset of women from each group.

There are a number of practical implications of these findings for service providers. The establishment of migrant and refugee women’s sexual and reproductive rights requires a combination of system improvements and services that benefit women (Sen & Govender, 2015). This includes acknowledgement of the specific needs of migrant and refugee women and the production of culturally safe health promotion strategies and sexual health resources (Botfield et al., 2016; Keygnaert et al., 2014). Sexual health promotion needs to be a key part of early resettlement for migrant and refugee women (McMichael & Gifford, 2009). More specifically, there is a need for recognition of potential lack of knowledge in young women about the association between menstruation and fertility, a need for provision of information for community workers on menstruation and menopause to facilitate education of migrant and refugee women, and support for women in educating their daughters about menstruation (Hawkey et al., 2016a). Misconceptions and absence of information about contraception need to be addressed, through the provision of resources and support in a range of modalities, in the language of different cultural groups, and husbands involved in the discussion (Ngum Chi Watts, McMichael, & Liamputtong, 2015). Difficulties experienced by unmarried women in obtaining information about contraception, and sex and sexual health need to be acknowledged (Salad et al., 2015). Understanding of the social and cultural sensitivities that many migrant and refugee women experience regarding discussion of sex or sexual concerns and the implications this has for sexual health and sexual rights will have benefits for provision of sexual health services, and understanding of sexual embodiment (Dean et al., 2017). Resources on sexual rights and consent, the value of pleasure and desire, female genital cutting, sexual infections and screening, how to discuss sexual concerns with a health professional, and how to avoid or treat sexual pain and discomfort, as well as sexuality education for young people, can address women’s unmet needs in this sphere (Helmer, Senior, Davison, & Vodic, 2015). Overall, sensitivity to language and other barriers to sexual health service use need to be improved, with previous research suggesting a need for bilingual community-based interpreters or “navigators” (Henderson & Kendall, 2011, p. 195).

In conclusion, this study demonstrated that migrant and refugee women’s sexual embodiment is experienced in the context of cultural discourses and practices, with implications for sexual knowledge, sexual behavior and sexual health. Women’s sexual subjectivity is constructed and reconstructed through social interactions and negotiation of competing discourses associated with sexual embodiment. Researchers and health service providers need to be aware of the cultural and religious constraints which may impede the development of migrant and refugee women’s agentic sexual subjectivity, but also their capacity for resistance and negotiation.

## Footnotes

1 The term “culturally and linguistically diverse” (CALD) is used by the Australian government to describe people who have a cultural heritage different from that of the majority of people from the dominant Anglo-Australian culture (Australian Government Department of Health, 2016), replacing the previously used term of people from a “non-English speaking background” (NESB). As this term is not used in Canada, we prefer the use of “migrant and refugee women” to describe the women who participated in the study.
